# Assessing the Influence of Patient Empowerment Gained Through Mental Health Apps on Patient Trust in the Health Care Provider and Patient Compliance With the Recommended Treatment: Cross-sectional Study

**DOI:** 10.2196/48182

**Published:** 2024-02-12

**Authors:** Julien François, Anne-Françoise Audrain-Pontevia, Sana Boudhraâ, Stéphane Vial

**Affiliations:** 1 École des Sciences de la Gestion Université du Québec à Montréal Montréal, QC Canada; 2 Centre de Recherche de l'Institut Universitaire en Santé Mentale de Montréal Montréal, QC Canada; 3 École de Design Université du Québec à Montréal Montréal, QC Canada

**Keywords:** patient empowerment, patient compliance, patient trust, mental health app, mental health

## Abstract

**Background:**

In chronic mental illness, noncompliance with treatment significantly worsens the illness course and outcomes for patients. Considering that nearly 1 billion people worldwide experience mental health issues, including 1 of 5 Canadians in any given year, finding tools to lower noncompliance in these populations is critical for health care systems. A promising avenue is apps that make mental health services more accessible to patients. However, little is known regarding the impact of the empowerment gained from mental health apps on patient compliance with recommended treatment.

**Objective:**

This study aimed to investigate the impact of patient empowerment gained through mental health apps on patient trust in the health care provider and patient compliance with the recommended treatment.

**Methods:**

A cross-sectional web-based survey was conducted in Canada. Eligible participants were Canadian adults diagnosed with chronic mental health disorders who were using at least one of the following apps: Dialogue, MindBeacon, Deprexis, Ginger, Talkspace, BetterHelp, MindStrong, Mindshift, Bloom, Headspace, and Calm. A total of 347 valid questionnaires were collected and analyzed using partial least-squares structural equation modeling. Trust in the health care provider and patient compliance were measured with multiple-item scales adapted from existing scales. Patient empowerment was conceived and measured as a higher-order construct encompassing the following 2 dimensions: patient process and patient outcome. All the items contributing to the constructs in the model were measured with 7-point Likert scales. The reliability and validity of the measurement model were assessed, and the path coefficients of the structural model were estimated.

**Results:**

The results clearly show that patient empowerment gained through mental health apps positively influenced patient trust in the health care provider (β=.306; *P*<.001). Patient trust in the health care provider had a positive effect on patient compliance (β=.725; *P*<.001). The direct relationship between patient empowerment and patient compliance was not significant (β=.061, *P*=.23). Interestingly, the data highlight that the effect of patient empowerment on patient compliance was fully mediated by trust in the health care provider (β=.222; *P*<.001). The results show that patient empowerment gained through the mental health app involves 2 dimensions: a process and an outcome.

**Conclusions:**

This study shows that for individuals living with mental health disorders, empowerment gained through mental health apps enhances trust in the health care provider. It reveals that patient empowerment impacts patient compliance but only through the full mediating effect of patient trust in the health care provider, indicating that patient trust is a critical variable to enhance patient compliance. Hence, our results confirm that health care systems could encourage the use of mental health apps to favor a climate that facilitates patients’ trust in health care provider recommendations, possibly leading to better compliance with the recommended treatment.

## Introduction

### Background

Defined as the extent to which a patient’s behavior coincides with the medical or health advice given by a health care provider (eg, psychiatrists, psychologists, nurses, clinical social workers, or case managers [1]), patient compliance plays a vital role in health care provider–patient relationships and health care systems [2]. However, noncompliance to treatment is still one of the greatest challenges in mental health care services, and how to improve compliance remains an issue [3]. The prevalence of noncompliance in mental health disorders is high; studies estimate that approximately 44% of patients with depression and anxiety [4], 50% of those with schizophrenia [5], and 68% of those with opioid use disorder [6] are not fully compliant with recommendations. The literature emphasizes that a lack of patient compliance leads to poor outcomes, which increase health care service use and overall health care costs [7]. For instance, for 2020, US $290 billion of avoidable health care costs have been attributed to noncompliance in the United States [8], and the cost was US $4 billion in Canada [9]. The global economic burden of mental health disorders is expected to rise to US $8.5 trillion in 2030 [10]. Additionally, mental health disorders are a real public health problem [11]. Today, nearly 1 billion people worldwide have mental health issues [12], including 1 of 5 Canadians in any given year [13]. Therefore, it has become a priority to engage patients with mental health disorders to actively participate in their care with the goal of improving the cost-effectiveness of care delivery [14]. This can be achieved with the help of IT, such as mental health apps [7], which improve access to care for patients at the time and place of user convenience at low cost [8]. Mental health apps have been found to empower patients with mental health disorders by actively engaging them in their treatment and in collaborating with their health care provider in the decision-making process [7]. This study examined the relationships between 2 predictors theoretically related to patient compliance among individuals living with mental health disorders using mental health apps. These predictors were patient empowerment and patient trust in health care providers, which have been identified as critical predictors of patient compliance [15-17]. Hence, the objective of this research was to identify the successive impact of empowerment on trust in the health care provider and patient compliance for mental health app users. By addressing this research objective, this study proposed corresponding theoretical and practical implications.

### Mental Health App Use

With the proliferation of digital devices and smartphones, health care systems are now implementing digital approaches to deliver mental health at scale [18]. e–Mental health refers to the set of information and communication technologies dedicated to supporting and enhancing the delivery of mental health care [19]. According to the Mental Health Commission of Canada [20], e–mental health services are an effective and complementary solution to traditional care. Today, e–mental health services are diverse and may include, but are not limited to, smartphone apps, web-based information portals, teleconsultation, and virtual reality devices.

In 2021, there were more than 20,000 mental health apps worldwide, and the global market for mental health apps had an annual growth rate of 20% [21]. Mental health apps are smartphone-delivered platforms that provide self-directed or remotely facilitated mental health services in the areas of communication, self-monitoring, diagnosis, and treatment [22]. These apps are designed and used for patients with major depressive disorders [23], bipolar disorders [24], anxiety disorders [17], substance abuse disorders [25], schizophrenia [26], and psychotic disorders [27]. Mental health apps enable their users to better understand their health conditions and allow them to engage with practices that would benefit their health [28]. These devices allow individuals to monitor their symptoms, track their treatment, learn about their health condition, or exchange information with their health care provider. While improving patient activation and facilitating communication with the health care provider, these apps facilitate patient adherence to the recommended treatment. Yet, the literature reveals that the effectiveness of mental health apps is difficult to assess and remains questioned [29]. Besides, research shows that the use of mental health apps dramatically drops over time, with a sustained use of these apps after 6 weeks varying from 0.5% to 28.6% [30]. In addition, the effectiveness of mental health apps is difficult to assess. As an example, there were over 10,000 mental health mobile apps available in the market in 2017; the effectiveness and quality of these services were questioned [26].

### Patient Empowerment

In the health care literature, the concept of patient empowerment was introduced in the early 1990s [31]. Since then, it has been the object of increasing attention from both scholars and practitioners [32]. Still, the health care literature reveals a lack of consensual definition [33] and highlights that patient empowerment can be defined through the lens of 2 distinct conceptual approaches.

A first conceptualization considers patient empowerment as a process of behavior change [34,35]. This approach finds its roots in the self-determination theory [36], which proposes that patients are self-determining agents who have the capacity for autonomy. In the context of mental health, the need for autonomy is achieved through patients’ involvement in managing their own health [37]. Patient involvement refers here to the importance patients attribute to their health in general and to the acts of maintaining their health [38]. It is manifested in the form of involvement in decision-making and active participation in consultation with health care providers [39]. In turn, active participation facilitates patients’ knowledge development [35]. This is consistent with Funnel et al [31], who emphasized that patients are empowered when they have sufficient knowledge to make rational decisions and have sufficient experience to evaluate the effectiveness of their decisions.

A second conceptualization of patient empowerment considers this construct as an outcome. Empowerment as an outcome refers to a sense of self-efficacy that occurs as a result of the process [34]. In this view, self-efficacy refers to a function of an individual’s abilities, knowledge, and learned skills to achieve a desired goal related to their health [40]. This results in an increased feeling of control for patients over their health condition [41]. This is in accordance with the findings of McAllister et al [14], who posited that patients are empowered when they have behavioral control, that is, when they are able to take action to reduce harm or improve their lives.

Considering the 2 abovementioned conceptualizations, patient empowerment is operationalized as a second-order construct formed by 2 first-order independent constructs: patient process and patient outcome. Specifically, we argue that the 2 patient empowerment dimensions are unique first-order constructs because they are independent. This is why patient empowerment is conceptualized as a reflective-formative second-order construct. Consistent with this, we hypothesize that (H1) patient process has a positive effect on empowerment and (H2) patient outcome has a positive effect on empowerment.

### Patient Trust in Health Care Providers

Mental health care providers are health care professionals, including psychiatrists, psychologists, nurses, clinical social workers, or case managers [1]. Research shows that empowerment can encourage patient trust in the health care provider [16]. Patient trust in the health care provider refers to the willingness of a patient to be vulnerable to the actions of a health care provider, based on the expectation that the health care provider will perform a particular action that is important to the patient, irrespective of the patient’s ability to monitor or control that health care provider [42]. Among the most commonly described dimensions of physician behavior on which patients are believed to base their trust are competence, compassion, privacy and confidentiality, reliability and dependability, and communication [43]. Patients’ comfort and confidence in taking control of their health care strengthen the level of trust and commitment they have with their health care provider [44]. Specifically, patient perceived control and participative communication were found to have direct positive effects on trust [45]. Consistent with this view, scholars have demonstrated that patient control over the chronic illness condition and patient participation during medical consultations positively impact patient trust in the health care provider [16]. On the basis of the rationale above, we believe that empowerment gained through mental health apps leads to greater trust in the health care provider. We therefore propose the following hypothesis: (H3) empowerment gained through mental health apps has a positive effect on trust in the health care provider.

### Patient Compliance

Patient compliance can be seen as an outcome of the relationship between the patient and the health care provider [46]. Patient compliance refers to the patient’s adherence to treatment recommendations and prescriptions targeted to his or her particular disease [47]. The literature reveals that patient compliance is alternatively conceptualized either as a process or as an outcome. Patient compliance as a process relates to the extent to which a patient’s behavior coincides with medical advice [48]. Compliance as an outcome is defined as the number of doses taken correctly, which supports the therapeutic outcome [48]. As patients engage in diverse behaviors to increase their well-being and health, and because health care providers give them the means to do so by offering recommendations, treatments, and guidance, it is expected that patient empowerment will enhance patient compliance. Therefore, we propose that (H4) empowerment has a positive effect on patient compliance. Lastly, research suggests different relationships between the patient and the health care provider. Specifically, scholars highlight that trust in the health care provider facilitates the patient to accept the actions of the health care provider [43], and that trust leads to better commitment to the recommended treatment [49]. Given that compliance requires the health care provider to give patients recommendations and prescriptions [48], it is expected that trust leads to greater patient compliance. On the basis of these considerations, we propose the following hypothesis: (H5) trust in the health care provider has a positive effect on patient compliance.

In summary, based on existing literature, the aim of the study is to investigate the impact of patient empowerment gained through mental health apps on patient trust in the health care provider and patient compliance with the recommended treatment.

## Methods

### Ethical Considerations

This study was approved by the ethics committee on research involving humans of the University of Québec in Montréal (2022-3559). Several specific ethical considerations were considered, and the following was implemented: First, the participants were informed of the length of the survey, the number of questions, the purpose of the study, and the university organizing data collection. Second, the survey did not include identifiable information, and all participants were anonymous. Third, respondents received an incentive after completing the survey, such as cash, airline miles, gift cards, and vouchers.

### Study Design

A cross-sectional web-based survey was conducted with Canadian adults (aged 18 years and older) living with a mental health disorder from May 2022 to July 2022. The self-reported survey was administered on the web-based survey platform Qualtrics. The survey is described according to the CHERRIES (Checklist for Reporting Results of Internet E-Surveys) checklist (Multimedia Appendix 1) [50].

### Data Collection

A total of 6 experts reviewed the clarity, writing style, and flow of the questionnaire and assessed the understandability of the items. This led to some minor adjustments in the wording of 3 items. The recruitment of the participants was done by Qualtrics, which used niche panels and randomly selected respondents according to inclusion criteria. Three inclusion criteria were defined while ensuring a representative sample of the sociodemographic characteristics of the Canadian population regarding age, sex, income, education, and race. First, we targeted Canadian adults who were using or had used at least one of the following mental health apps: Dialogue, MindBeacon, Deprexis, Ginger, Talkspace, BetterHelp, MindStrong, Mindshift, Bloom, Headspace, and Calm. This selection is based on the judgment of 2 mental health experts who recommended 2 selection criteria: estimated number of users and availability to the patients on the market. Second, we selected participants diagnosed with a mental health disorder. We offered participants the opportunity to indicate the mental health disorder they were experiencing from the following list, which included the most prevalent mental health disorders in Canada: anxiety, bipolarity, depression, schizophrenia, and posttraumatic stress. Additionally, we gave participants the opportunity to specify their mental health disorder in case it was not identified in the abovementioned list. Third, we chose participants who spoke either French or English. To ensure that the participants met the abovementioned inclusion criteria, self-reported questions were included in the questionnaire.

To determine the minimum sample size, the “10 times the number of structural paths directed at a particular construct in the structural model” rule of thumb was assessed [51]. Accordingly, the minimum sample size for the model of this research is 50 participants because the number of structural paths in the research model is 5. A total of 347 surveys were collected, which is high above the minimum required. Table 1 presents the statistics that describe the participants and their characteristics. Of the participants, 46.1% (160/347) were male, 49.6% (172/347) were female, and 4.3% (15/347) were gender-fluid, nonbinary, or two-spirit. In terms of age distribution, the age groups 26-35 years (112/347, 32.3%) and 36-49 years (94/347, 27.1%) were the most represented. Education level was relatively high, with university level accounting for 46.1% (160/347). The annual income distribution was relatively even. Participants had been diagnosed with a mental health disorder (ie, depression, anxiety, schizophrenia, and posttraumatic stress) and were using or had used at least 1 mental health app. The users in our study mainly use the apps Calm (82/347, 23.6%) and Headspace (60/347, 17.2%). Participants were also or had been under the care of a health care provider.

**Table 1:** The demographics of the sample (N=347).

Category			Value
**Sex, n (%)**			
	Male	160 (46.1)	
	Female	172 (49.6)	
	Gender-fluid, nonbinary, or two-spirit	15 (4.3)	
**Age (years), n (%)**			
	18-25	89 (25.6)	
	26-35	112 (32.3)	
	36-49	94 (27.1)	
	50-65	48 (13.8)	
	>66	4 (1.2)	
**Educational** **background**			
	Postsecondary	114 (32.9)	
	Secondary	68 (19.6)	
	University	160 (46.1)	
	Prefer not to answer	5 (1.4)	
**Income (US $)**			
	<39,999	89 (25.6)	
	40,000-79,999	114 (32.9)	
	80,000-119,999	72 (20.7)	
	120,000-159,999	32 (9.2)	
	160,000-199,999	27 (7.8)	
	>200,000	13 (3.7)	

### Scale Development

The scales used in the measurement model were designed by the researchers based on the existing literature. The scale of patient process included 1 item to measure health involvement adapted from Oh and Lee’s [52] scale and 3 items to measure knowledge development adapted from the scale by Prigge et al [15]. The scale for patient outcome included 1 item to measure the patient’s feeling of control adapted from Oh and Lee’s scale [52] and 3 items to measure self-efficacy adapted from the scale by Prigge et al [15]. Items used to measure patient process and patient outcome were adapted to the context of mental health app use behavior. Patient empowerment is conceptualized as a construct constituted by 2 independent dimensions: patient process and patient outcome. Therefore, we operationalized patient empowerment as a second-order construct. We chose the repeated indicators approach to measure this construct as the approach is suitable for measuring a higher-order construct [53]. We used this approach instead of the 2-stage approach or the hybrid approach [53] because patient empowerment is measured as a formative-reflective second-order construct [54] and was an endogenous construct in the path model [53]. Consequently, we used the manifest variables of the first-order latent variables (patient process and patient outcome) to measure the second-order latent variable (patient empowerment). The trust scale included 5 items based on the scale by Anderson and Dedrick [55]. Finally, the patient compliance scale consisted of 2 items based on the scale by Prigge et al [15]. We used age, sex, education, and income as control variables; they were each measured with a single item. The questionnaire used 7-point Likert scales anchored from “completely disagree” to “completely agree” (Multimedia Appendix 2 [15,52,53,55]). As the Likert scales we used included more than 5 categories, these scales were treated as continuous measures. This follows the literature recommendations [56-58] and meets the partial least-squares (PLS) structural requirements [53,59].

### Data Analysis

The data were analyzed by PLS structural equation modeling (PLS-SEM). SEM is considered a second-generation multivariate analysis technique that allows researchers to incorporate unobservable or latent variables measured indirectly by observable variables [60]. PLS-SEM was chosen instead of covariance-based SEM as it does not assume that data are distributed normally, as was the case with our data [51]. The measurement model (the inner model) was first assessed; we evaluated the reliability and validity of the estimates for the latent variables. The structural model (the outer model) was then calculated to assess the direction and significance of the relationships between the latent variables [60,61]. To do so, we used the SmartPLS 3 software (SmartPLS GmbH) for the PLS-SEM analysis. We chose the standard bootstrap procedure on 5000 bootstrapping samples to examine the structural model’s path coefficients and the corresponding significance levels [62].

## Results

### Measurement Model (Inner Model)

Because all the constructs were reflectively measured in our model, assessing the measurement model was required to evaluate the reliability, the convergent validity, and the discriminant validity of the measurement scales [51]. Table 2 shows the composite reliability, Cronbach α, and average variance extracted (AVE). The factor indicators, known as the outer loadings or reflexive indicator loadings, were superior to the 0.5 threshold recommended for each indicator, demonstrating that the chosen indicators adequately measure the latent variables [51]. The Cronbach α coefficient values ranged from .762 to .863, and the composite reliability values were between 0.770 and 0.868, indicating that constructs had good reliability. All the average variances extracted were greater than 0.5, suggesting a good convergent validity for the 5 constructs. Altogether, these findings demonstrated that the 5 constructs had good convergent validity.

To establish the discriminant validity, the square root of the AVE of each construct had to be larger than its correlation with other constructs. Additionally, because empowerment was operationalized as a second-order construct, we do not consider the discriminant validity between both patient outcome and patient process and their higher-order component patient empowerment. The correlation between patient outcome (patient process) and patient empowerment is expected to be greater than the square root of the AVE of patient outcome (patient process) because the measurement model of the patient empowerment construct repeats the indicators of its 2 lower-order components (patient process and patient outcome) [53]. Table 3 shows that the square root of each factor’s AVE value is greater than the other factor correlation coefficients, indicating a good discriminant validity for the 5 constructs.

**Table 2:** Construct reliability and convergent validity.

Construct and item		Indicator loading	Cronbach α	Composite reliability	Average variance extracted
**PC^a^**			.851	0.851	0.870
	PC1	0.934	N/A^b^	N/A	N/A
	PC2	0.932	N/A	N/A	N/A
**PP^c^**			.816	0.834	0.651
	PP1	0.826	N/A	N/A	N/A
	PP2	0.871	N/A	N/A	N/A
	PP3	0.870	N/A	N/A	N/A
	PP4	0.637	N/A	N/A	N/A
**PO^d^**			.762	0.770	0.651
	PO1	0.872	N/A	N/A	N/A
	PO2	0.829	N/A	N/A	N/A
	PO3	0.767	N/A	N/A	N/A
**PT^e^**			.863	0.868	0.646
	PT1	0.866	N/A	N/A	N/A
	PT2	0.797	N/A	N/A	N/A
	PT3	0.773	N/A	N/A	N/A
	PT4	0.767	N/A	N/A	N/A
	PT5	0.812	N/A	N/A	N/A
**Patient empowerment**			.843	0.850	0.520
	PP1	0.750	N/A	N/A	N/A
	PP2	0.816	N/A	N/A	N/A
	PP3	0.780	N/A	N/A	N/A
	PP4	0.597	N/A	N/A	N/A
	PO1	0.760	N/A	N/A	N/A
	PO2	0.663	N/A	N/A	N/A
	PO3	0.656	N/A	N/A	N/A

^a^PC: patient compliance.

^b^N/A: not applicable.

^c^PP: patient process.

^d^PO: patient outcome.

^e^PT: patient trust in the health care provider.

**Table 3:** Construct discriminant validity.

Construct	PC^a^	PE^b^	PO^c^	PP^d^	PT^e^
PC	0.933	N/A^f^	N/A	N/A	N/A
PE	0.283	0.721	N/A	N/A	N/A
PO	0.278	0.844	0.824	N/A	N/A
PP	0.230	0.918	0.561	0.807	N/A
PT	0.744	0.306	0.278	0.265	0.804

^a^PC: patient compliance.

^b^PE: patient empowerment.

^c^PO: patient outcome.

^d^PP: patient process.

^e^PT: patient trust in the health care provider.

^f^N/A: not applicable.

### Structural Model (Outer Model)

To assess the structural model for collinearity issues, we considered the variance inflation factor in the predictor constructs as indicative of collinearity [60]. In this study, all variance inflation factor values were below 5, which indicates no violation of the multicollinearity assumption. The model’s goodness of fit was our criterion to assess the overall fit of the model. The model’s goodness of fit for this study was 0.607, allowing us to conclude that our model performed well [63]. The effects of the control variables—age, sex, education, and income—on patient trust in the health care provider have been found to be insignificant.

**Figure 1:**
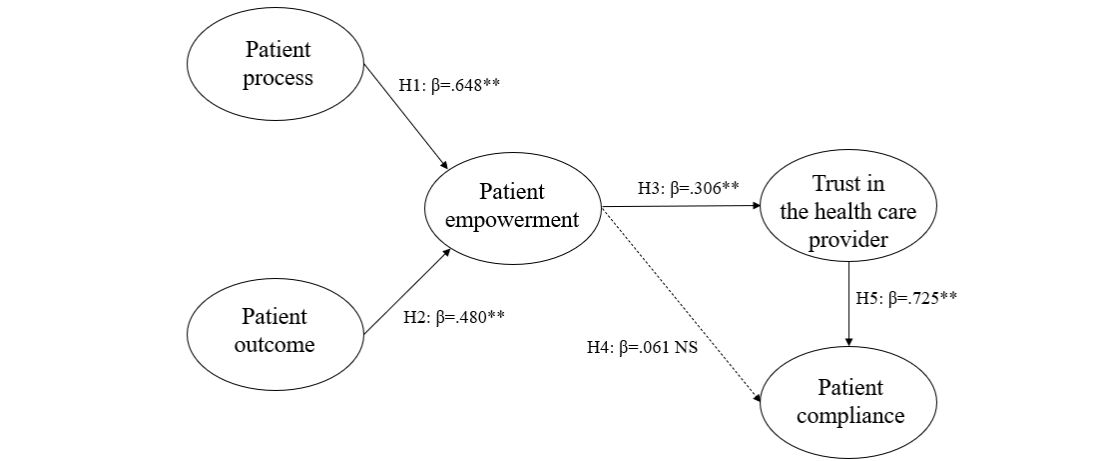
Structural model showing path coefficients (β). **P<.001. NS: not significant.

The results showed that all the hypothesized anticipated relationships are supported except for the impact of empowerment on patient compliance (Figure 1). Four (H1, H2, H3, and H5) of our 5 hypotheses were validated by our data (Table 4). Patient process was found to be a first-order construct of empowerment as patient process positively impacted patient empowerment (β=.648; *P*<.001). Similarly, patient outcome was found to be a first-order construct of empowerment as patient outcome positively impacted patient empowerment (β=.480; *P*<.001). Besides, patient empowerment was found to have a positive effect on patient trust in the health care provider (β=.306; *P*<.001). Patient trust in the health care provider also had a positive effect on patient compliance (β=.725; *P*<.001). Interestingly, the relationship between empowerment and patient compliance was not significant (β=.061, *P*=.23).

We conducted the indirect effect (mediation) of trust to further understand the relationship between empowerment and patient compliance. The indirect effect via the path empowerment→trust→patient compliance significantly mediated the effect of empowerment on patient compliance (β=.222; *P*<.001), indicating that trust fully mediated the relationship between empowerment and patient compliance (Table 5).

**Table 4:** Hypothesis test results.

Hypothesis	Path	Path coefficient (SD)	*P* value	Result
H1	PP^a^→PE^b^	0.648 (0.023)	<.001	Supported
H2	PO^c^→PE	0.480(0.021)	<.001	Supported
H3	PE→PT^d^	0.306 (0.062)	<.001	Supported
H4	PE→PC^e^	0.061 (0.051)	.23	Rejected but fully mediated by trust in the health care provider
H5	PT→PC	0.725 (0.042)	<.001	Supported

^a^PP: patient process.

^b^PE: patient empowerment.

^c^PO: patient outcome.

^d^PT: patient trust in the health care provider.

^e^PC: patient compliance.

**Table 5:** The mediation effect of trust in the health care provider.

Path	Direct effect			Indirect effect	
	β value	*P* value	β value		*P* value
PE^a^→PT^b^→PC^c^	.061	.23	.222		<.001

^a^PE: patient empowerment.

^b^PT: patient trust in the health care provider.

^c^PC: patient compliance.

## Discussion

### Principal Findings

The objective of this study was to investigate the impact of patient empowerment gained through mental health apps on patient trust in the health care provider and patient compliance. We examined the 2 dimensions of patient empowerment and its effects on trust in the health care provider and patient compliance. Data were collected from participants with mental health disorders using the survey method and analyzed with the PLS-SEM method. Findings reveal that patient empowerment is a second-order construct composed of 2 dimensions: patient process and patient outcome. The data revealed that patient empowerment directly and positively impacts patient trust in the health care provider. Patient trust in the health care provider also directly and positively impacts patient compliance. However, no direct effect of patient empowerment on patient compliance was supported, indicating that trust is a full mediator between patient empowerment and patient compliance.

### Theoretical Contributions

This study contributes to the research field in 4 ways. First, this research clearly shows that patient empowerment gained through mental health apps enhances patient compliance. Previous research that has shown a positive relationship between patient empowerment and patient compliance has been conducted on patients living with chronic physical diseases [15,64]. While focusing on individuals living with mental health disorders, this study is the first to demonstrate the effect of patient empowerment on compliance with the recommended treatment in mental health app users.

Second and importantly, this study reveals that the relationship between the patient empowerment gained through the mental health app and patient compliance is fully mediated by patient trust in the health care provider, indicating that patient trust is a critical variable to enhance patient compliance. Past research has found that patients in a hospital setting who were empowered through education and doctor support demonstrated more trust in their caregivers [44], and that patient trust in the health care provider enhances patient compliance [2,65]. Our study supports these findings in the field of mental health. It shows the existence of a positive relationship between patient empowerment gained through an app dedicated to mental health and patient trust in the health care provider. In turn, our findings highlight that patient trust significantly enhances patient compliance with the health care provider recommendations. While showing that patient empowerment gained using a mental health app has no direct effect on compliance with the health care provider’s recommended treatment, but that it increases patient trust, this study underlines that, thanks to mental health apps, patient empowerment and patient trust are intertwined to enhance patient compliance. Hence, this research provides a better understanding of the cognitive mechanisms and paths through which mental health apps enhance user compliance with the caregiver’s recommendations.

Third, incidentally, this study provides an interesting theoretical view, operationalization, and validation of the construct of patient empowerment. We propose a thorough conceptualization and measurement of this construct. The literature in health care remains inconclusive and reveals a lack of consensus in defining patient empowerment [33,35,66,67]. We propose that the patient empowerment gained using mental health apps be a multidimensional construct, built around 2 dimensions. Precisely, we define patient empowerment as a patient’s sense of control in managing their health status, resulting from (1) an increase in patient knowledge and (2) a voluntary commitment to improving their health. In line with previous studies [30,34], this definition conceives that patient empowerment is a second-order construct, consisting of both a process and an outcome. The outcome dimension refers to the feeling of control and the patient’s ability to self-manage, whereas the process dimension refers to knowledge development and self-involvement in managing their health.

### Practical Contributions

These findings have several implications for practice. First, the results suggest that health care providers should encourage and educate individuals living with mental health disorders to adopt and use mental health apps. The use of mental health apps would be beneficial given that the number of individuals with mental health disorders is increasing, especially young people. This research demonstrated that empowerment gained by a mental health app has a positive effect on patient trust in the health care provider. Hence, health care providers should play a crucial role in the adoption of mental health apps for their users. They should educate patients, engage in discussions with them, and answer their questions and concerns to facilitate the adoption process of mental health apps. This education process is important because the misuse of such devices can harm patients who would conduct incorrect self-diagnosis due to poor eHealth literacy and misinterpreted information [68]. Health care providers sometimes lack knowledge and skills regarding health app use [69]; as a result, this process also implies that health care providers should be trained to recommend and support mental health apps. Health authorities and mental health app designers or specialists appear to be the right entities to train health care providers.

Second, human-centered designers, alongside software developers, should further develop features that empower the patients, for example, functionalities that improve the patients’ self-efficacy or knowledge development or that support and stimulate self-management strategies [70]. Designers and developers could, for instance, develop mental health apps that feature alarms, reminder scheduling, behavioral tracking tools, symptoms manager, care pathway timeline, a system of conversations with peers, automated routines, and healthy lifestyle daily advice. This should be done through a co-design process rooted in a human-centered design [71] that allows end users (patients) to meaningfully contribute to the definition of these features as closely as possible to their lived experience.

Third, this study suggests that, because patients are compliant when they trust their health care provider, these providers should create a climate that facilitates patients’ trust in their practices. For instance, health care providers should consider patients’ needs and concerns, answer their questions with respect for their feelings, and demonstrate empathy.

### Limitations

Although this study enriches the knowledge of patient empowerment using mental health apps and its effects on patient-health care provider relationships, it has several limitations. The first limitation regards the use of a cross-sectional survey, which reflects data from respondents at one point in time. Hence, it is recommended to carry out a longitudinal survey in the future to extend the validity of our findings. Second, our model focused on 3 key variables: patient empowerment gained using mental health apps, patient trust in the health care provider, and patient compliance. Further research investigating the effect of patient empowerment on other variables, such as patient commitment, patient satisfaction, or patient social support, should improve our knowledge of the effects of the use of mental health apps. Third, participants in our sample were individuals living with mental health disorders in Canada, which offers a universal health care system to its citizens. It would be of interest to test the proposed theoretical framework on other countries having different health care systems, such as private health care systems. This would increase the external validity of our findings. Fourth, investigating the effect of mental health apps for each disorder pathology in isolation would enrich the present findings. After this study, we will conduct a second research project to examine case by case the effects of mental health apps across various mental health disorders such as depression, anxiety, or bipolarity. The fifth limitation of this study relies in the self-report assessment of the mental health diagnosis. Yet, self-report assessment for mental health diagnosis was found to be highly and significantly correlated to clinical assessments [72]. Conducting an observational study in the future would complement these findings and thus extend the validity of our results.

### Conclusions

This study clearly shows that mental health apps enhance patient empowerment for an individual living with mental health disorders, which in turn leads to patient trust in the health care provider. Interestingly, patient empowerment gained using mental health apps is found to impact patient compliance, but only through the full mediating effect of patient trust in the health care provider. This research suggests that health care providers should encourage patients to use mental health apps to enhance their feeling of empowerment. We believe that this research stream is and shall continue to be of considerable interest as empowering patients has become a priority for policymakers, with the goal of improving the quality and efficacy the health care delivery system.

## Appendix

Multimedia Appendix 1 Checklist for Reporting Results of Internet E-Surveys (CHERRIES).

Multimedia Appendix 2 Measurement instruments.

## Abbreviations

AVE: average variance extracted
CHERRIES: Checklist for Reporting Results of Internet E-Surveys
PLS-SEM: partial least-squares structural equation modeling

## Data Availability

The data sets generated during and/or analyzed during this study are available from the corresponding author on reasonable request.
