# Supplementary material for: Assessing the Influence of Patient Empowerment Gained Through Mental Health Apps on Patient Trust in the Health Care Provider and Patient Compliance With the Recommended Treatment: Cross-sectional Study
Source: J Med Internet Res. 2024 Feb 12;26:e48182. doi: 10.2196/48182 (PMC10897799; doi:10.2196/48182)
Supplement: Multimedia Appendix 1 [file jmir_v26i1e48182_app1.docx]

**Multimedia Appendix 1: Checklist for Reporting Results of Internet E-Surveys (CHERRIES)**

| **Item category** | **Checklist item** |  |
| --- | --- | --- |
| Design | Describe survey design | Cross-sectional study (N=347).  The target population was Canadian adults (18 years old and older) living with a mental health disorder.  The self-reported survey was administered on the Web-based survey platform Qualtrics. |
| IRB (Institutional Review Board) approval and informed consent process | IRB approval | This study was approved by the Ethics Committee on Research Involving Humans of University of Québec in Montréal (2022-3559). |
|  | Informed consent | The participants were informed of the length of  the survey, the number of questions, the purpose of the study, and the university organizing the data collection. |
|  | Data protection | The survey did not include identifiable information, and all participants were anonymous. |
| Development and pre-testing | Development and testing | - Questionnaire development: - The scales used in the measurement model were designed by the researchers based on the existing literature. - 6 experts reviewed the clarity, writing style and flow of the questionnaire and assessed the understandability of the items. This led to some minor adjustments in the wording of 3 items. |
| Recruitment process and description of the sample having access to the questionnaire | Open survey versus closed survey | Closed surveys. All surveys could only be accessed by invitation. |
|  | Contact mode | Initial contact with potential participants was made via email. |
|  | Advertising the survey | Not applicable. |
| Survey administration | Web/E-mail | The survey was administered using Qualtrics, with a link to access the survey supplied by email. |
|  | Context | Not applicable. |
|  | Mandatory/voluntary | Not applicable. |
|  | Incentives | Respondents received an incentive after completing the survey, such as cash, airline miles, gift cards, and vouchers. |
|  | Time/Date | From May 2022 to July 2022 |
|  | Randomization of items or questionnaire | The items or questionnaires were not randomized. |
|  | Adaptive questioning | Adaptive questioning was not applied. |
|  | Number of Items | The questionnaire had 56 items. |
|  | Number of screens (pages) | The survey had 7 online pages. |
|  | Completeness check | A completeness check was carried out after the survey  was closed. Only complete responses (responses from participants who had answered the whole survey) were used. |
|  | Review step | The respondents were able to change their answers using a Back button. After completing the questionnaire and submitting the data, data could not be changed by the participants. |
| Response rates | Unique site visitor | Not applicable. |
|  | View rate (Ratio unique site visitors/unique survey visitors) | Not applicable. |
|  | Participation rate (Ratio unique survey page visitors/agreed to participate) | Not applicable. |
|  | Completion rate (Ratio agreed to participate/finished survey) | There were no incomplete surveys. |
| Preventing multiple entries from the same individual | Cookies used | Not applicable. |
|  | IP check | Not applicable. |
|  | Log file analysis | Not applicable. |
|  | Registration | The participants received a unique identification code and accessed the online survey by invitation via email. |
| Analysis | Handling of incomplete questionnaires | Not applicable. |
|  | Questionnaires submitted with an atypical timestamp | Not applicable. |
|  | Statistical correction | Not applicable. |

Adopted from Eysenbach [50].
