# Supplementary material for: Assessing the Influence of Patient Empowerment Gained Through Mental Health Apps on Patient Trust in the Health Care Provider and Patient Compliance With the Recommended Treatment: Cross-sectional Study
Source: J Med Internet Res. 2024 Feb 12;26:e48182. doi: 10.2196/48182 (PMC10897799; doi:10.2196/48182)
Supplement: Multimedia Appendix 2 [file jmir_v26i1e48182_app2.doc]

**Multimedia Appendix 2:** Measurement instruments.

| **Construct** | **Scale/Scoring** | **Items** | **Source** |
| --- | --- | --- | --- |
| Patient process | - 7-point Likert - Strongly disagree to strongly agree | - Using this app or website I would say that I feel better informed than other people with the same disorder. - Using this app or website I would say that I gain knowledge about my mental health status. - Using this app or website I would say that I understand my mental health better. - Using this app or website I would say that I can motivate myself to manage my mental health and make a better life. | [14,52] |
| Patient outcome | - 7-point Likert - Strongly disagree to strongly agree | - Using this app or website I would say that I can better manage a minor problem without having to consult. - Using this app or website I would say that I can usually think of a solution if I am in trouble. - Using this app or website I would say that whatever happens to my health, I can handle it. | [14,52] |
| Patient empowerment | - 7-point Likert - Strongly disagree to strongly agree | Items from patient process and patient outcome constructs (repeated indicators approach [53]) | [14,52] |
| Trust in the health care provider | - 7-point Likert - Strongly disagree to strongly agree | - I trust him or her so much that I always try to follow their advice. - He or she is a true expert in the management of my mental health. - If he or she tells me something is true, then it must be true. - I count on him or her to let me know if a mistake has been made in his or her recommendations. - He or she is usually attentive to my needs and puts them first. | [55] |
| Patient compliance | - 7-point Likert - Strongly disagree to strongly agree | - I mostly follow his/her recommendations. - I follow his/her recommendations on a regular and continuous basis. | [14] |
